# Understanding Reactions to Informative Process Model Interventions: Ambivalence as a Mechanism of Change

**DOI:** 10.3390/bs14121152

**Published:** 2024-12-02

**Authors:** Nimrod Rosler, Ori Wiener-Blotner, Orel Heskiau Micheles, Keren Sharvit

**Affiliations:** 1 Program in Conflict Resolution and Mediation, Tel Aviv University, Tel Aviv 6997801, Israel; 2 Department of Psychology, University of Haifa, Haifa 3103301, Israel

**Keywords:** psychology of conflict, conflict-supporting narratives, cognitive ambivalence, emotional ambivalence, attitude change, decisional balance

## Abstract

Transforming the course of protracted and bloody conflicts requires changing the behaviors and minds of society members who take part in these conflicts. While studies examining the psychology of such societies point to the barriers that conflict-supporting narratives create for changing minds and behavior, a novel psychological intervention offers a new direction to facilitate openness for attitude change based on the Information Process Model (IPM). Previous studies indicated the effectiveness of this intervention in creating an unfreezing of conflict attitudes and increasing support for peace negotiation in different conflict areas. However, since the psychological process underlying its effectiveness remains underexplored, the aim of the current research is to examine the experiences of participants exposed to IPM-based messages and the role of cognitive and emotional ambivalence in facilitating the unfreezing of conflict-supporting narrative and contemplating alternative beliefs. The first study (n = 234) examines how IPM (vs. control) videos increase engagement with and ambivalence towards conflict-supporting narratives using quantitative and qualitative analysis of written Decisional Balance responses. The second study (n = 24) delves into the expressions of cognitive and emotional ambivalence following exposure to different segments of an IPM video using semi-structured interviews, and further assesses their potential influence on facilitating contemplation with newly provided information.

## 1. Introduction

The attack of 7 October 2023 on Israel and the following war in Gaza has taken an unprecedented toll on Israelis and Palestinians, shifting world attention once again to their ongoing conflict and to the immense human tragedy created by its continuation. Surveys carried out among Israelis and Palestinians (e.g., [1,2,3]) since then reflect the intensity of beliefs legitimizing the atrocities committed against the rival and record-low levels of support for negotiation and peaceful resolution of the conflict. Taken together, they highlight the pressing need to change the behaviors and minds of society members who take part in protracted and bloody conflicts to transform the course of these conflicts.

Past studies pointed to the challenges of changing deep-rooted beliefs and attitudes prevalent among society members in such conflicts, taking the form of shared coherent narratives [4]. Consequently, scholars who study the psychological aspects of protracted and bloody conflicts, sometimes referred to as intractable conflicts, suggested interventions aimed at overcoming these narratives to create attitude and behavior change [5]. While some of these interventions were found to be effective in creating attitude change, little attention has been given to their mechanism of influence [6]. The question that therefore remains is how do psychological interventions affect the cognitions and emotions of society members so that they are willing to consider changing their long-held set of beliefs about the conflict?

In the current paper, we explore the reactions of individuals exposed to a recently introduced psychological intervention for facilitating change of conflict-supporting narratives in intractable conflicts—the Informative Process Model (IPM; [7]). We examine whether exposure to messages following the IPM leads to cognitive and emotional ambivalence in order to understand how such messages facilitate the unfreezing of conflict-supporting narratives and contemplation of alternative beliefs.

### 1.1. Conflict-Supporting Narratives and Interventions to Change Them

Societies engulfed by intractable conflicts (Intractable conflicts are characterized as violent and protracted conflicts that demand extensive investment and play a central role in the lives of individuals in the involved societies, and are perceived by those affected as total, irresolvable, and of a zero-sum nature [8,9,10]) live for prolonged periods under conditions of intense threats, deprivation of needs, stress, hardship, and resource austerity. To adapt to the difficult conditions and cope with the challenges that the conflicts create, members of these societies form collective narratives [8,11,12]. These narratives include societal beliefs referring, among other themes, to the justness of the ingroup’s goals, the importance of security, delegitimization of the rivals, ingroup glorification and victimization, and yearning for peace [8,13,14,15,16,17,18].

The developed narratives become hegemonic, widely shared, and deeply entrenched since they serve the basic needs jeopardized by conflict [4,19,20]. Given their importance, these narratives are constantly reinforced in society through processes of social influence which creates *cognitive freezing* [21,22,23]: a preference for maintaining the narratives and resisting their change, leading to the rejection of alternative information and perspectives [24], even when opportunities for peace arise [25].

Previous psychological interventions to facilitate change and promote peaceful attitudes and behaviors were found to be effective under specific conditions, and to varying degrees, with each intervention having its own limitation (see [26]). In the context of intractable conflicts, many of these interventions do not take into account the full scale of psychological dynamics, with the deep-rooted narratives, and the unfulfilled needs that underlie individuals’ resistance to changing their dysfunctional and costly behaviors (e.g., [4,27]). To meet these challenges and limitations, we recently introduced a novel intervention—the *Informative Process Model* (IPM; [7]).

### 1.2. The Informative Process Model (IPM) and the Role of Ambivalence in Change

The IPM was developed to facilitate the unfreezing of conflict-supporting attitudes and beliefs in the context of intractable conflicts. The IPM maintains that explaining how and why conflict-supporting narratives develop in intractable conflicts, and the role they play in maintaining the conflict, while also suggesting the possibility of alternatives, can lead to unfreezing of these narratives and readiness to consider alternative attitudes. Specifically, an IPM-based intervention includes the following four elements: (1) clarifying that conflict-supporting narratives evolve to fulfill the needs of society members involved in intractable conflicts; (2) explaining that these narratives are common among all societies involved in such conflicts; (3) adding that these narratives come with a cost of fueling the conflict and describing the immense costs to society that come with it; and (4) suggesting that there is a benefit to exploring alternative means of fulfilling these needs, as found in other peacefully-resolved conflicts, which may end the cycle of violence and proceed to peacemaking.

Drawing on the literature from both attitude change and clinical psychotherapy (see [28]), the IPM is based on an acceptance–change dialectic, where one is exposed to messages that humanize and normalize conflict-supporting narratives, while simultaneously challenging them to consider a less costly alternative. The first two elements of the IPM-based message legitimize currently held beliefs and attitudes of most individuals living in societies in intractable conflict, thereby acknowledging them as cogent. The latter two elements convey a message of change, suggesting that there may be alternatives that meet the same needs as the previously held attitudes and beliefs but without their costs. The purpose of the IPM’s acceptance–change dialectic is to invite a sense of *ambivalence* that, in turn, encourages unfreezing and compels individuals to reassess their current attitudes and beliefs.

Ambivalence has been defined as a psychological state in which an individual’s distribution of considerations includes inconsistent or contradictory beliefs, emotions, or evaluations to a certain degree [29,30,31]. Therefore, ambivalence can serve as an important and necessary marker of the extent to which someone simultaneously considers multiple beliefs or experiences multiple emotions, whether it involves one’s original belief and emotion or new adopted alternatives. It seems to have an important part to play in destabilizing existing beliefs and influencing individuals’ willingness to explore alternative ones and is often the natural and necessary intervention needed for change [32,33,34,35,36].

Ambivalence is elicited by IPM-based interventions in a way that is meant to address key causes of attitudinal rigidity relating to intractable conflicts, such that individuals are afforded an opportunity to seek methods of coping with the costs of intractable conflicts without perpetuating existing conflict-supporting narratives. By offering validation and legitimacy to perspectives supporting the conflict narrative and those opposing it, the ambivalence-inducing intervention invites the reduction of strong emotions, which are often exacerbated by attitudinal expression among individuals with similar views [37]. Furthermore, by first acknowledging the conflict-supporting narrative, but then adding context to those experiences, the IPM makes room for new attitudes and emotions besides the pre-existing ones. Since IPM-based interventions have mostly been tested for effectiveness in changing attitudes, the current paper aims at empirically exploring the role of ambivalence as one of the main *mechanisms* for facilitating change.

### 1.3. Attitude Change in Intractable Conflicts: Outcomes and Mechanisms

Models of attitude change in social psychology predominantly focus on attitude change as an outcome, where interventions measure certain attitudes prior to, and following an intervention, or compare change among experimental vs. control conditions to determine whether attitudinal change occurred or not (e.g., [38]). While this approach enables empirical demonstration of the effectiveness of specific interventions, it has its limitations. It is especially limiting in the context of intractable conflicts, where attitudes are particularly rigid, and attitudinal change is most often an effortful, costly, conscious, and gradual process [39]. Therefore, in addition to understanding *what* changes attitudes and beliefs, it is important to better understand *how* the changing of those attitudes and beliefs occurs.

Previous experimental studies of IPM-based interventions provided empirical support for their effectiveness in facilitating unfreezing and change in the context of the Israeli–Palestinian conflict. Specifically, intervention videos (vs. control) were found to increase deliberation of new information, which predicted acceptance of the IPM-based message, which, in turn, predicted support for negotiations [7]. This effect was found when using messages focusing on different themes of conflict-supporting narratives, such as delegitimization of the rival group, justness of own-group goals, security and victimhood [7,28]. Similar to previous psychological interventions, past studies mostly focused on the outcomes of change rather than on the mechanisms leading to it.

Only one study of IPM-based interventions examined a particular psychological mechanism, *feeling accepted*, which is inherent to the model ([28], Study 3). Specifically, we found that an IPM-based intervention increased feelings of acceptance among study participants, which, in turn, led to greater deliberation of new information, predicting greater reconsideration of attitudes concerning the Israeli–Palestinian conflict. While these findings empirically substantiate the theoretical basis of the acceptance–change dialectic that stands at the basis of the intervention, other psychological mechanisms that underlie its effects require further unpacking. The *Transtheoretical Model* of intentional change (TTM) provides a complementary and relevant theoretical framework for filling gaps in our understanding of the mechanisms behind the effects of IPM-based interventions, since it identifies change as a gradual process, rather than a final outcome [40].

The TTM highlights the importance of understanding shifts in attitudes, beliefs, and behaviors, particularly in response to adverse experiences [41]. While based in clinical psychology, the principles of the TTM have been effectively applied to understand and facilitate change in diverse contexts and areas of studies (e.g., [42,43,44,45]). According to the TTM, change is often not an immediate shift from one attitude to another, but rather occurs as a gradual process that requires reflection and reevaluation, hence taking time and effort and involving several stages. Specifically, the model suggests that change occurs in a progression of five stages and identifies what is most effective in facilitating transition between them [46,47]. Each stage delineates varying relationships individuals have with their attitudes, beliefs, and behaviors, and presents tasks required to move to the next stage [41,48]. In the context of intractable conflicts, and with the purpose of unfreezing conflict-supporting attitudes and behaviors, the most relevant transition is between the first two stages of precontemplation and contemplation.

### 1.4. The Transition Between Precontemplation and Contemplation in Intractable Conflicts

The first stage in the TTM, *precontemplation*, is where people do not perceive any costs to their attitudes or problems with their behavior and its consequences, and therefore deny or reject the need to make changes; alternatively, they have become demoralized and see no reason to try and change [36]. The second stage, *contemplation*, categorizes people who perceive their behavior as problematic, actively consider the advantages and disadvantages of changing, and are in a state of ambivalence (The remaining three stages in the model are *preparation***,** when people decide to change and begin taking small steps towards making those changes in their lives; *action*, when individuals successfully modify their environment, perceptions, or behaviors to overcome their problematic behavior for an extended period; and *maintenance*, when individuals work to consolidate the gains they have attained, and work to prevent relapse for an intermittent period [49]). Given that conflict-supporting narratives are deeply entrenched and those holding them are usually unaware of the costs of maintaining them, it is likely that most members of society involved in intractable conflict are in the precontemplation stage. Furthermore, even those who have wishful aspirations for conflict resolution, but feel that there is nothing they can do about it realistically [50], can be defined as precontemplators. Since precontemplators across contexts are resistant to recognizing the problem or might not have any intrinsic motivation to explore change, psychological interventions aimed at advancing peacemaking should focus on the transition from precontemplation to contemplation.

The stage of contemplation in intractable conflicts could be described as understanding that changing attitudes and behaviors relating to the conflict has merit. This is the stage where the unfreezing of conflict-related attitudes would occur, since individuals are neither committed to their previously held attitudes and beliefs, nor to new ones. Accordingly, the individual is not ready to act based on new attitudes, either because existing attitudes and beliefs are still very dominant, or the alternative ones are not quite established yet. The transition from precontemplation to contemplation does not imply that a shift in attitudes has already occurred, but it is a significant and necessary step towards lasting change. The goal of the IPM-based intervention is to facilitate unfreezing through the experience of ambivalence, where society members are actively weighing the costs and benefits of their existing and alternative attitudes and beliefs, which reflects contemplation. Therefore, the goal of IPM-based interventions can be said to help people to transition from precontemplation to contemplation by facilitating ambivalence.

### 1.5. The Present Research

The aim of the present research is to explore the cognitive and emotional mechanisms that underlie the change process initiated by IPM-based interventions. Because ambivalence seems to play an important role in destabilizing existing beliefs and influencing individuals’ willingness to explore alternative attitudes and behaviors, participants’ reported ambivalence can be informative as to the impact that an intervention has had on them. As in previous IPM studies [7,28], we focus on the Israeli–Palestinian conflict which is considered as a prototypical intractable conflict. We therefore conducted two studies, both prior to the recent Israel–Hamas war, examining the extent to which IPM-based interventions elicit ambivalence and how it is reflected in Jewish–Israeli participants’ reactions to the intervention. In Study 1, by taking mixed quantitative and qualitative approaches, we attempt to gauge the early stages of contemplation among participants through their reported ambivalence regarding intractable conflicts after watching IPM-based videos using an open-ended questionnaire. In Study 2, we deepen our exploration of ambivalence by conducting semi-structured interviews with participants while they are watching IPM videos, enabling us to follow their cognitive and emotional reactions to the intervention step by step. The studies were carried out in parallel with each other, creating a convergent design of mixed methods research [51]. While the method and results of each study will be presented separately, they will be discussed together in the last section, taking a simultaneous bidirectional approach to their integration [52].

## 2. Study 1

Since the TTM is new to the field of intractable conflicts and may prove a valuable tool to inform researchers of participants’ readiness to change or unfreeze attitudes, Study 1 aimed to examine how an IPM-based intervention enables the transition between precontemplation and contemplation through attitudinal ambivalence. Accordingly, we use an adapted version of the *decisional balance* measure, based on the TTM, as an indicator of cognitive ambivalence.

The decisional balance measures individuals’ ambivalence and overall attitude towards change by having them weigh the pros and cons of present attitudes and behaviors and of changing them, where responses vary across the different stages of change. Since change requires the weighing of potential benefits and losses associated with the alternative, this measure can also serve as a predictor of future behavior change [36]. Longitudinal studies demonstrate the potential of implementing this index to inform interventions that compel transitions across stages of change (e.g., [53,54]). Specifically, Prochaska and his colleagues [41] found that participants’ reports of the pros of changing their behavior were higher in the contemplation stage than in the precontemplation stage, suggesting that the progress across those two stages requires increased perceived benefits of change. Similarly, while cons of changing behavior outweighed the pros in precontemplation, the pros and cons were more balanced in contemplation [41]. Our research hypothesis was that participants exposed to an IPM-based message would report a higher ratio of perceived benefits to perceived costs of changing their opinions than the control group.

### 2.1. Method

#### 2.1.1. Participants

Two hundred forty-five Israeli Jews were recruited through the research participation system of a university in northern Israel and through student-associated social media groups. Six participants were excluded for failing to answer one of two attention-check questions after watching the IPM video, and four others for responding to questions in a way that suggests that they did not understand the instructions in the study. Out of the remaining 235 participants (180 female, 55 male, M_age_ =25.97, SD_age_ = 5.03), 51.5% identified as rightists, 17.4% as centrists, and 31.1% as leftists when considering political ideology. Participants were paid 30 NIS (equivalent to $8) or granted partial course credit for their participation.

#### 2.1.2. Procedure and Materials

The study was conducted online. Participants were randomly assigned into an IPM-based intervention group or a control group. The intervention group watched a combination of four short IPM-based video-clips in Hebrew, similar to the ones used in previous research by Rosler et al. ([7]; see https://youtu.be/PDeshDBVT9g (accessed on 1 October 2024)), each following the theoretical underpinnings of the IPM, and tailor-made for this purpose in collaboration with a professional video producer. The videos all share a similar structure, opening with a partially hidden figure and quotes that are relatable with conflict supporting narratives that Israelis may have heard, or believe themselves. Then the character is revealed to be someone who experienced an unrelated intractable conflict. This part of the message stresses the normative nature of these narratives. After showing that the character is from another conflict, the message explains that the narrative is natural and even deemed necessary to effectively cope with the conditions of conflicts, thus validating conflict-supporting narratives.

The last part of the video suggests that conflict-supporting narratives perpetuate the conflict, and that change and conflict resolution are possible, by showing that, despite these beliefs, the character’s conflict came to a peaceful resolution. The video therefore proceeds by asking if there is another way and answering that there is a different way to cope with the conflict while indicating the peaceful alternative that took place in that conflict. The control group watched videos of generic commercials unrelated to conflicts.

After watching the videos, participants were asked four attention verification questions to ensure they were actively watching the videos. If the participants failed to answer the video attention checks correctly, they were notified and allowed to watch the videos one more time. If they failed to answer them correctly a second time, the study was terminated. Demographic characteristics of the participants were collected at the end of the study, including sex, age, place of birth, place of birth of parents, level of education, religious identification, and political orientation (Further measures were also assessed in the study but are not analyzed in the present research due to its scope and focus on exploring ambivalence. These include deliberation of new information, support for negotiation, and acceptance of IPM-based message).

#### 2.1.3. Measures and Analytical Framework

*Decisional Balance* was assessed using four open ended questions that ask participants to list the pros and cons of changing and maintaining their currently held beliefs regarding the Israeli–Palestinian conflict (see Table S1). Responses to the prompt of reasons for changing attitudes and beliefs, and the prompt of reasons against maintaining attitudes and beliefs were categorized as favoring changing attitudes and beliefs. Responses to the prompt of reasons against changing attitudes and beliefs, and the prompt of reasons for maintaining attitudes and beliefs were categorized as favoring maintaining existing attitudes and beliefs.

The content provided by participants in their responses was analyzed to shed light on the processes of attitudinal and belief change. The second author and three additional judges reviewed the 1696 participant responses and, blind to any information about the participants, including assigned condition, demographic information, and political affiliations, thematically coded the responses based on their content with a bottom-up approach. First, the second author reviewed a random selection of 20% of the responses and coded them based on the content of the responses, identifying the primary theme(s) and subject matter, and types of motivation. To refine the coding scheme, themes that appeared infrequently or proved challenging to consistently apply across responses were eliminated. All judges discussed the final themes to ensure there was an agreement and mutual understanding of the themes, and then they independently reviewed the randomly selected 20% of the responses. After completing 20% of the responses, the judges convened, discussed, and compared how they coded the responses. In cases of disagreement among the judges regarding the appropriate themes of a given response, they engaged in thorough discussions until mutual agreement was reached for that response and for future responses like it. Afterwards, the judges reviewed and coded the rest of the responses. Kappa analysis was conducted to determine interrater reliability (average score, 0.8; see Table S2), in accordance with best practices [55], following which the judges discussed coding differences again until a final agreement was reached (The study also attempted to validate multiple-choice quantitative measures of the TTM’s decisional balance, specifically a measure based on the work of Janis & Mann [56] and a single item measure based on Velicer et al. [54], adapted to the context of intractable conflict. However, we decided not to use these measures due to concerns about the construct validity of the modified versions).

### 2.2. Results

#### 2.2.1. Assessing Impact of IPM-Based Intervention on Decisional Balance

**Figure 1:**
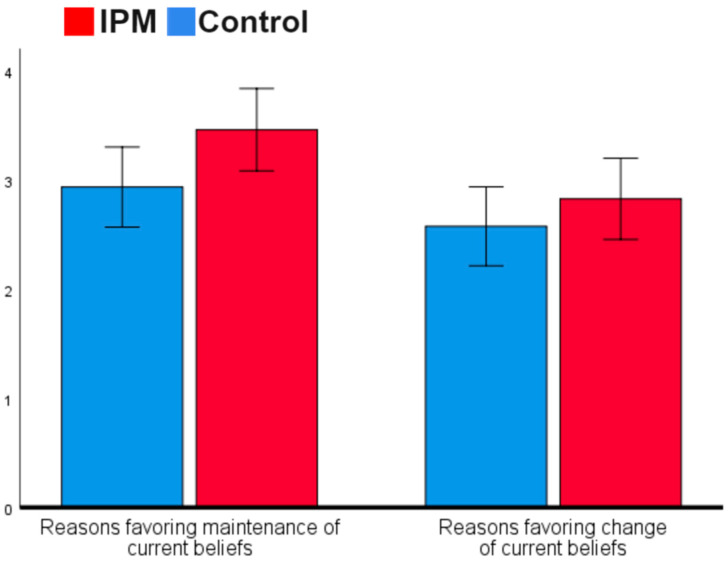
Mean number of reasons to change and maintain attitudes and beliefs by experimental condition in Study 1.

To determine if the IPM-based message impacted decisional balance responses, we conducted a mixed model ANOVA (2 × 2) with a condition (IPM vs. control) as a between-subject factor, the type of responses listed in the open ended questions (i.e., reasons for changing vs. for maintaining current views regarding the Israeli–Palestinian conflict) as a within-subject factor, and the number of responses in each category as the dependent variable (see Figure 1). The analysis revealed that participants in both conditions reported more reasons for maintaining their attitudes and beliefs than changing them, F(1, 233) = 5.53, *p* = 0.020, η^2^ = 0.023. Participants in the IPM condition listed more reasons for changing and maintaining existing attitudes and beliefs than the control group, F(1, 233) = 4.33, *p* = 0.039, partial η^2^ = 0.018, and the interaction was not significant, F(1, 233) = 1.380, *p* = 0.241, partial η^2^ = 0.006.

#### 2.2.2. Qualitative Analysis of Considerations in Decisional Balance Measure

**Figure 2:**
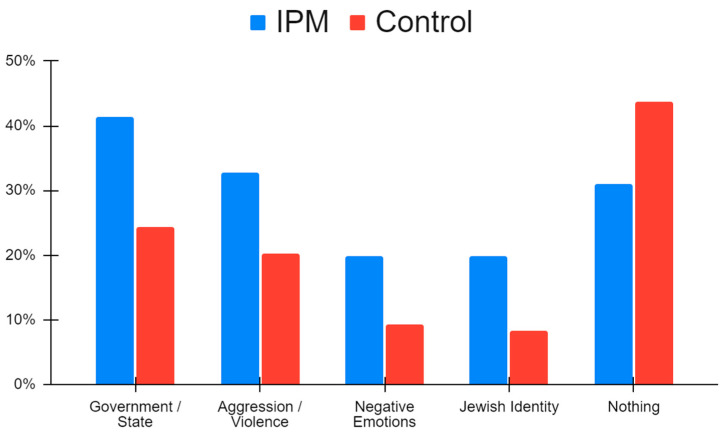
Differences in thematic content between conditions from Study 1.

We wanted to determine if participants’ considerations for changing or maintaining existing attitudes and beliefs were affected by the IPM-based intervention. Since there were more responses from the IPM condition, we first controlled for response count by dummy coding each theme, where participants were given a “1” if the theme was present in any of their responses, and a “0” if the theme was not present in any of their responses. We then conducted chi-square tests to determine whether the prevalence of themes differed between experimental conditions. Participants in the IPM condition more often referenced the themes of Government/Statehood, Jewish Identity, Aggression/Violence, and Negative Emotions than participants in the control group (see Table S3) (For an additional method of measuring the effects of the IPM-based intervention on participant decisional balance, where participant responses are measured by the number of coded themes in each response out of their total amount of thematic codes, see Table S4. Results from this additional method were largely similar to those reported here). The only theme that was more prevalent among control group participants was responses that conveyed participants not having any considerations for changing and/or maintaining their current attitudes and beliefs (e.g., “Nothing”). In most of these cases, there were no statistically significant thematic differences across reasons for changing or maintaining existing attitudes and beliefs, except for two cases: Jewish Identity was more likely to be mentioned in the IPM condition as a reason to maintain existing attitudes and beliefs (χ^2^ = 5.22, df = 1, *p* = 0.035); and the Government/Statehood theme was both more prevalent in participant responses in the IPM condition (χ^2^ = 6.29, df = 1, *p* = 0.017), as well as more often mentioned as a reason to maintain existing attitudes and beliefs (χ^2^ = 10.07, df = 1, *p* = 0.002; See Figure 2; for thematic examples of responses for changing or maintaining beliefs, see Table S5).

**Figure 3:**
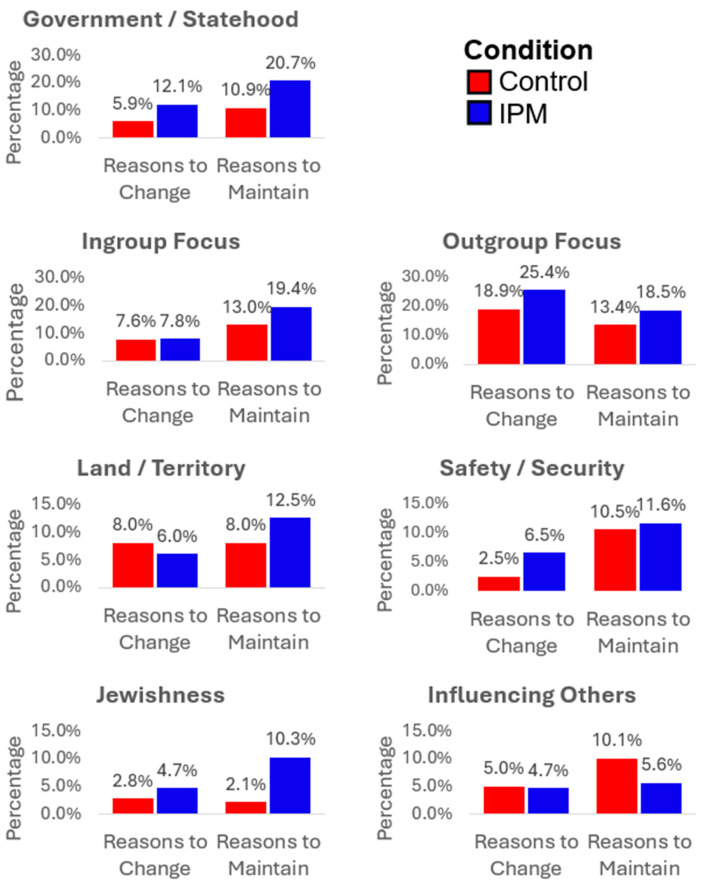
Percentages of Thematic Occurrences as Reasons to Change or Reasons to Maintain Existing Conflict-Related Attitudes and Beliefs Across Conditions in Study 1.

Among the remaining themes, Land/Territory was more likely to be mentioned as a reason to maintain existing beliefs in the IPM condition (χ^2^ = 5.77, df = 1, *p* = 0.016); influencing other people’s opinions was more often mentioned as a reason to maintain attitudes and beliefs in the control condition (χ^2^ = 4.33, df = 1, *p* = 0.038); outgroup-focused reasoning was more likely a reason for change in the control group and for maintaining in the IPM condition (χ^2^ = 5.80, df = 1, *p* = 0.016); ingroup-focused reasoning was more likely mentioned as a reason for maintaining attitudes and beliefs in both the IPM condition (χ^2^ = 13.40, df = 1, *p* < 0.000) and the control condition (χ^2^ = 3.85, df = 1, *p* = 0.050), but even more so in the IPM condition than in the control condition (χ^2^ = 16.22, df = 1, *p* < 0.00; see Figure 3). The remaining differences between conditions were not significant.

### 2.3. Discussion

Study 1 aimed to assess the effect of the IPM-based intervention on attitudinal ambivalence, as assessed through the decisional balance among Jewish–Israeli participants. The results of the study indicated that the IPM intervention had a significant impact on participants’ responses to the Decisional Balance measure. Participants in the IPM condition listed significantly more reasons for changing and for maintaining their current beliefs about the Israeli–Palestinian conflict compared to the control condition. This result in the IPM condition is in line with the characteristics of someone in the contemplation stage, since individuals in the contemplation stage are more active in weighing the merits and costs of their circumstances [36]. This suggests that the IPM-based intervention led to increased contemplation and consideration of multiple perspectives regarding attitudes and beliefs about the conflict. Furthermore, the majority of content themes in responses to the Decisional Balance appeared equally as considerations for maintaining and changing existing beliefs. This balanced representation of themes as both pros and cons suggests that the IPM-based intervention can be effective in encouraging participants to think critically and holistically about their attitudes and beliefs. This finding supports the efficacy of the IPM in promoting a more balanced consideration of one’s own beliefs, including its costs and benefits.

The content analysis of the decisional balance responses not only suggests that the IPM-based intervention facilitates more contemplation than in the control condition (in the quantity of responses), it also facilitates more substantive and diverse content-related considerations than in the control group, going beyond the content presented in the IPM-based videos. For example, the consideration of one’s Jewish identity and the implications thereof, which were not a part of the content of the videos at all but came up in participants’ responses (e.g., “My children could lose their Jewish identity”), suggests that participants were not just addressing considerations thematically related to the IPM-based video’s content, but were applying the messages to themes that were relevant to them.

At the same time, participants in the control condition were more likely to not participate in the Decisional Balance task, by saying they had nothing to gain or lose, or that they had nothing to consider that would influence what they thought, said, or did. Furthermore, beyond apparent lower levels of engagement, influencing others’ attitudes and beliefs was most often considered by control participants as a reason for maintaining existing attitudes, the opposite of reconsidering their own attitudes. This attitude, where one either ignores existing issues or is resistant to changing them, is consistent with the precontemplation stage of change. Therefore, the difference between conditions in readiness to explore the costs and benefits of various attitudes and beliefs suggests that the IPM effectively increased the likelihood that participants were in the contemplation stage. Finally, results also showed that many of the themes appeared in similar frequency in both conditions, which suggests that certain themes are generally common considerations for existing and alternative attitudes and beliefs, such as peace, needs for security, and distribution of territory.

To further explore the expressions of attitudinal as well as emotional ambivalence among participants watching IPM-based videos, we conducted in-depth interviews in Study 2. These interviews aimed at providing detailed understanding of the cognitive and affective effects allowing transition to *contemplation* by each part of the intervention—the parts pertaining to *acceptance* of current-held beliefs, and the one relating to *change* in them.

## 3. Study 2

The aim of Study 2 was to identify and thoroughly analyze not only the cognitive experiences of participants watching IPM-based videos, but also the emotional ones. Alongside the cognitive aspects of conflict-supporting narratives, negative emotions also act as major psychological barriers that inhibit progress towards peaceful resolution and contribute to narrative freezing [4]. Fear and anger are considered two of these impactful emotions in this context [57].

*Fear* is based on the evaluation of a situation as threatening and is associated with a sense of uncertainty and helplessness [58,59]. It has been identified as the dominant emotion among societies engulfed by intractable conflicts, since it fulfills functional roles: it creates permanent readiness for potential dangers, focuses attention on signs and information of possible threats, and increases cohesion, solidarity, and mobilization against the external threat by the rival [60,61,62]. However, interpreting every statement or action by the rival group as threatening reinforces the existing societal beliefs about victimhood, siege mentality, and mistrust, and leads to increased closure of the mind to avoid further risks [63].

Unlike fear, *anger* derives from the appraisal of a harmful act as unjustified and predicts actively taking action to correct the perceived wrongdoing [64,65]. Since violent acts by the rival—perceived as illegitimate—are one of the characteristics of intractable conflicts, anger is one of the most common emotions in such contexts [57]. While anger can increase certainty in currently held cognitions, it may also lead to greater willingness to consider alternatives to the current situation and support political change, if those are perceived as advancing correction of the wrongdoing [66,67].

To address negative emotions arising due to conflict experiences and the cognitive rigidity established by conflict-supporting narrative, scholars of intractable conflicts have pointed to the pivotal role of promoting *hope* [68]. Hope involves a wish for a positive outcome in the future and an expectation to achieve it, as well as positive affect about the desired outcome [69,70,71,72]. It enables planning and setting goals, cognitive flexibility, and creativity [60,70,71,73]. Hope has been found to increase support for peace negotiation and compromises [50,74,75] in contexts of intractable conflicts, and therefore various psychological interventions have been aimed at promoting hope (e.g., [76,77,78]).

Specifically, the first step of the IPM-based intervention, in which the psychological challenges that the conflict experiences create are reiterated, may give rise to common negative emotions in this context, such as fear and anger. Acknowledging their normalcy can later create a sense of relief, while the last element of the IPM suggesting alternative beliefs that allowed peacemaking in other cases may create hope. In this way, the different elements of the IPM can create *emotional ambivalence* as well.

The video used in Study 2 focused on the theme of *delegitimization* of the rival, since this theme, dominant on both sides of intractable conflicts, is widely used in the Jewish–Israeli discourse regarding Palestinians and their narrative (see [79,80]). Delegitimization excludes the rivals from the sphere of human groups and provides psychological permission to harm them and to reject them as partners for negotiations [81,82,83], and hence serves as one of the major barriers to peacemaking [84]. Using qualitative research methods, we examine the emotional and cognitive processes that participants undergo in each part of the intervention manipulation, in order to assess their impact on participants’ belief systems and attitudes.

### 3.1. Method

#### 3.1.1. Participants

Jewish–Israeli participants were recruited through student-associated social media groups of a university in central Israel. One hundred and twenty-eight people who responded to the ad were asked to complete a socio-demographic questionnaire, and a follow-up telephone conversation was conducted with them to briefly explain the technical conduct of the interview using a recorded Zoom video meeting and examine their willingness to participate. Those who were not students, those reporting no political involvement whatsoever, and those who were not able to participate in a recorded Zoom video meeting were dropped from the sample. Another follow-up email was sent to schedule the interview, and 35 participants responded, out of which 11 changed their minds or canceled (The interviews were carried out when the COVID-19 pandemic was widespread, leading to a relatively high drop-out rate). The 24 participants we interviewed (12 female, 12 male) had a mean age of 26. As for political identification, six identified as rightists, five as moderate right, six as centrists, five as moderate left, and two as leftists. Participants were paid 25 NIS (equivalent to $6.7) for their participation, and interviews averaged 30 min in length.

#### 3.1.2. Procedure and Materials

After receiving the written consent of each participant, the interview was conducted by a Zoom video meeting and was recorded. At the beginning of the interview, the participant was welcomed and then watched one IPM-based video-clip in Hebrew, similar to the ones used in the previous study [7], focusing on the delegitimization theme from the conflict-supporting narrative (see second video at https://www.youtube.com/watch?v=PDeshDBVT9g (accessed on 1 October 2024)). Unlike previous studies, the video was split into three segments in order to closely track the experiences of participants in every part of the acceptance–change dialectic approach that the IPM follows. After watching each of the segments, the video was stopped, and the participant was interviewed according to the interview protocol that appears in Table S6. The video was split as follows:

1. The *first segment*, about 17 s long, presents the partially hidden figure and narrative-acknowledging quotes, and ends before revealing the figure, its identity or its context. This part is intended to normalize the conflict-supporting narrative and create identification with the hidden character.
2. The *second segment*, about 13 s long, begins with revealing the face of the figure and his identity as a French soldier who refers, in the previous quotes, to the French–Algerian war. It is followed by messages validating the normality of the narrative and its effectiveness in coping with the experiences of intractable conflicts. The first and second segments convey the *acceptance* element of the dialectic approach.
3. The *third segment*, about 13 s long, starts with the question “is there another way?”, followed by messages suggesting that an alternative narrative can lead to resolving the conflict, as in the case of the French–Algerian war that ended with a peace deal between the parties. The last segment completes the *change* element of the dialectic approach.

Each segment was analyzed separately, and the participant was asked about the emotions and thoughts, if any, elicited by that segment of the video, followed by a discussion on the topic. As noted, some of the questions were predetermined, while others were added in response to the dynamics that emerged during the interview and in alignment with the participant’s remarks at that point. In the final stage, after viewing all the segments, the participant was shown the entire video from start to finish without interruption, followed by a concluding discussion. At the end of this discussion, the interviewee was thanked and compensated. Each interview was fully transcribed for research purposes.

#### 3.1.3. Analytic Framework

**Figure 4:**
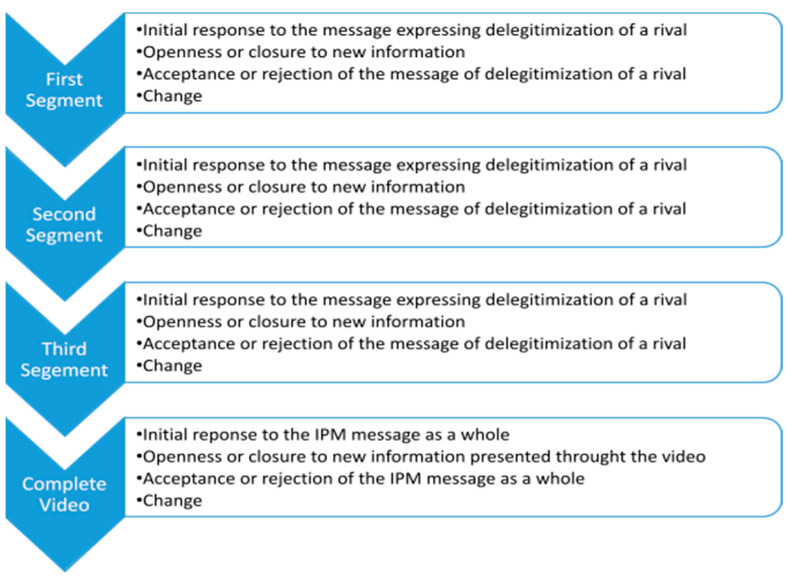
Stages of analysis of Study 2.

To understand the significance of the collected data, the findings from each interview were meticulously read and analyzed. The data were then organized separately and specifically according to each segment (i.e., the three segments and the segment where the entire video was shown). Each interview was analyzed according to the elements of the IPM (see Figure 4) as follows:

1. *Initial Response to the message* was examined both emotionally, i.e., how the participants felt after viewing the material, and cognitively, i.e., what thoughts arose following the viewing or their analytical processing of the information.
2. *Openness or Closure to new information* was assessed based on whether the participants were willing to consider and reflect on the new information presented to them, i.e., if they were open to listening to the information or chose to ignore it.
3. *Acceptance or Rejection of the message* was evaluated based on the degree of agreement with the message as stated by the participants, while also considering the level of interest, the way the information was processed, and the depth of engagement or immediate dismissal of the message.
4. *Change* occurring after viewing the segment or video was examined through the lens of the TTM. Specifically, we assessed whether the intervention prompted future-oriented thinking and whether the new information introduced new perspectives on viewing the conflict as resolvable, even if the interviewee has not yet committed to acting on this change in the near future, as described in the *contemplation* stage of the TTM.

The interviews were analyzed by the third author. Another independent judge analyzed a randomly selected 20% of the interviews according to the analytic framework. After completing their analysis of 20% of the interviews, the judges convened and compared how they coded the interviews. In cases of disagreement, they discussed those until a mutual agreement was reached for that interview and for future ones. Afterwards, the first judge analyzed the rest of the interviews.

Following the detailed analysis of each interview, the findings were organized to allow for a comprehensive review of all the interviews conducted. This approach aimed to systematically understand the participants’ responses to the video and to examine the degree of cognitive and emotional ambivalence it elicited. Accordingly, all participants’ responses to each segment were analyzed according to the model, in order to identify any recurring patterns or similar responses across different participants. To further confirm the patterns identified in the qualitative analysis through across-method triangulation (Triangulation refers to the use of multiple theories, data sources, or methods in qualitative research to develop a comprehensive understanding of the observed phenomena [85]) (see [86,87]), we used AntConc 4.3.1 software (see [88]). The software conducted a simplified statistical–comparative analysis to examine the recurrence of words among the interviewees across the various segments, while applying a 5 n-gram span to examine recurrence of relevant clusters (e.g., “discomfort” which is composed of two words in Hebrew) and to validate correct meaning of the words/clusters (e.g., “hope” and not “no hope”).

### 3.2. Findings

The findings of the analysis of the interview will be presented according to the different segments of the video. Results of the statistical analysis conducted using AntConc are presented in the relevant segment.

#### 3.2.1. First Segment

In this segment of the video, participants were presented with various quotes that introduced the theme of “delegitimization”, without any reference to the Israeli–Palestinian conflict. However, in their *Initial Response*, the vast majority of participants immediately connected the brief information they received to that conflict. Many participants noted that the message strongly resonated with memories of violent periods in the Israeli–Palestinian conflict, where such quotes were common, and they had to deal with difficult situations that created significant dissonance between the desire for peace and the harsh reality. For example, Interviewee No. 24 mentioned:

*I have flashbacks of suicides and attacks, and also situations I know from the army, where you suspect someone and feel uncomfortable […] but it turns out that this person had a knife, and he could have murdered a family.*

Interestingly, an analysis using AntConc revealed that the word “Arab” was mentioned by 18 different participants, and the word “Palestinian” was mentioned by 4 participants.

Additionally, the vast majority expressed some degree of aversion to the screened sentences at this stage, some because they found the generalization unreasonable, and others due to ambivalent feelings of both reservation and identification. Emotionally, most participants described a general sense of discomfort and unease when hearing the sentences in the first segment of the video. For instance, Interviewee No. 5 stated that she felt “*a certain discomfort, after all, it’s a really harsh phrasing, maybe I would have phrased it differently, but there were also things that were true*”.

This expression of discomfort moderated the anger towards the other side for some, due to the understanding that such statements could also be made about the ingroup, while, for others, it intensified the anger towards the adversary by supporting these messages. Accordingly, at this stage, the range of emotions described in response to this segment was negative, with some participants struggling to pinpoint a specific emotion, while others described being overwhelmed with feelings of anger, sadness, and despair. These findings are consistent with the literature on emotions in intractable conflicts, that highlight negative emotions as common in this context (see [57]). An analysis using AntConc showed that the words “discomfort” and “unease” were mentioned by seven different participants, and five referred to anger. It seems that the sense of discomfort facilitated the beginning of unfreezing through reservations, even if partial, regarding the phrasing and generalization of the statements.

In terms of *Openness or Closure* to new information, although no context was provided for the presented sentences in this segment, many participants found them familiar and noted that it was not the first time they had heard such statements. Nevertheless, some participants expressed varying degrees of interest and openness to the quotes. For some, the interest stemmed from perceiving the sentences as too extreme and a desire to oppose them, while for others, it arose from identification with their content. For example, Interviewee No. 11 said:

*[…] during the last [conflict] operation, I’m not talking about the part in Gaza, but about what happened here inside the country, the trust relations were quite eroded, so there can be a certain degree, like in certain moments, there can be a certain degree of identification.*

Accordingly, an analysis using AntConc showed that the word “extreme” was mentioned by 10 different participants, and the word “identification” was mentioned by 8 participants. Therefore, merging these findings with those of Study 1, we conclude that the way the sentences were presented without any context encouraged some participants to engage in a deeper analysis of the message, leading them to *contemplation* without judging the underlying intent. Since these messages were not new to most participants, those who chose to analyze the sentences in depth did so based on their existing set of beliefs while applying the sentences occurring in the video to content relevant to them, similar to our findings in Study 1.

Interestingly, when examining the degree of *Acceptance or Rejection* of the message, many participants described ambivalent feelings towards the content. On the one hand, they understood and accepted the message and could identify with it to some extent, but at the same time, some rejected the generalization made within it. All this occurred through creating an automatic connection to the Israeli–Palestinian conflict. Therefore, it is difficult to determine at this stage whether they accepted or rejected the message, and it appears that, in general, no significant *Change* occurred among the participants as a result of watching the first segment.

#### 3.2.2. Second Segment

When the figure of the French soldier is revealed, and it becomes clear that the quotes from the previous segment pertain to the French–Algerian war, there is a noticeable shift in the *Initial Response* of some participants. Some described a sense of relief and happiness, stemming from the fact that in the first segment, they believed the statements were about the Israeli–Palestinian conflict, leading to feelings of discomfort or anger. Upon realizing that the video actually concerns a different conflict, they felt somewhat reassured. For example, Interviewee No. 10 stated that she was “*glad it’s not something of ours*”. However, for about half of the participants, their feelings remained unchanged, and they continued to express discomfort. One of them noted, “*It doesn’t matter who says it... I pay less attention to who said it, but rather to the content, and I identify less with it*” (Interviewee No. 5).

Additionally, nine participants mentioned that the anger they felt, which was primarily directed at Israeli society, slightly diminished. For instance, Interviewee No. 15 said she felt “*maybe less anger, like an understanding that it’s not just here... so maybe in a way, it sort of softens, it’s like a kind of realization that it’s not just here*”. In other words, many at this stage experienced a process of normalization and acceptance of their emotions, which allowed them to openly identify with the statements expressing delegitimization of the opponent presented in the previous segment. As one participant noted, the second segment “*kind of confirms that what I’m feeling is natural... the sense of identification is more accepted, like it’s also okay to think this way, like it’s a kind of survival mechanism that protects us*” (Interviewee No. 12).

Finally, some participants found this segment to be a source of *hope*, even before watching the third segment. For instance, Interviewee No. 8 said:

*Maybe it makes me feel a bit more hopeful, because if it’s something that happens elsewhere and somehow in other places, they managed to resolve it in some way and achieve peace, then maybe our situation could be solvable too, and eventually, it will work out.*

Regarding *Openness or Closure* to new information, 13 participants were surprised and showed great interest and curiosity regarding the revelation of the figure and the focus on a different conflict than the one they initially thought. This allowed them to reassess the statements from the previous segment and their associated emotions, and even led some of them to examine whether the use of the delegitimization theme is indeed natural. Accordingly, some participants noted there is more room for such statements, as well as for the fear and anger they provoke, because they express legitimate feelings of different people in similar situations worldwide. This may be interpreted as granting these participants a preliminary sense of acceptance, which was previously found to facilitate greater unfreezing of conflict-related attitudes [28]. For example, Interviewee No. 15 stated:

*The exposure of the character made me see that this happens to everyone. I mean, everyone has a side, our side is good compared to your side, which is bad, but for me, the connotation immediately arose because it happens to them, I immediately associate it with what happens here, the Israeli–Palestinian conflict.*

Conversely, 11 participants remained closed off to the new information, and it did not affect their thinking regarding the use of the delegitimization theme, i.e., whether they identified with it or opposed it. It seems that the source of this closure was the fact that it concerns a conflict they are not personally involved in, and therefore does not create much interest. Interviewee No. 9 stated that “*obviously if it were an Israeli person or someone I know or don’t know, it doesn’t matter, someone from my country, then it would certainly be closer to my heart than a French or Algerian person with whom I have less connection*”. Additionally, for some, the closure arose from their rejection of the delegitimization of the opponent, seeing no justification for it even if it occurs in the context of another conflict. For example, Interviewee No. 1 noted, “*I knew that everywhere they would say about the enemy that he is this and that, and yes, they generalize, but that doesn’t mean it’s okay*”.

Regarding *Acceptance or Rejection* of the message, most participants indeed accepted the message, each for their reasons and to varying degrees. Some fully accepted the message after a process of normalization and justification of what was said. However, there were a few who described ambivalence towards the message, choosing to reject it in part, yet still accepting it to some extent. Interviewee No. 6 noted:

*Not all of them want to behave like beasts, some behave exactly the opposite, on the one hand. On the other hand, if we only look at the part that doesn’t want war, then we miss the part that behaves like beasts, so it’s natural but to a certain extent.*

It seems that many participants who rejected the previous message but did not do so decisively felt more comfortable accepting the message and identifying with it, understanding that intractable conflicts worldwide are quite similar in nature. For example, Interviewee No. 12 noted that she “*understands that it’s not maybe something that only happens here, like it’s something universal that happens, when you have enemies, you turn them into something inhuman*”.

On the other hand, eight participants rejected the message. Although they disagreed with the generalization it was based on, it seems that they understood the reason behind what was said and, to some extent, could identify with it more than after watching the first segment. For example, Interviewee No. 3 stated that he now understands better what was said and explained that “*it just became more believable and deeper, that I can maybe understand, not connect, just understand the person, who says that it has become legitimate to view the enemy as if he is not human*”.

As for *Change*, among the participants who accepted the new message after rejecting it in the previous segment, there was a certain shift in their perception, and some adopted more moderate positions. For instance, some participants who previously opposed the delegitimization of the opponent could now understand more deeply the societal need to use this narrative and therefore could identify with what was said. It appears that many felt more comfortable expressing their honest opinion without reservations after watching this segment, so it can be said that there was a change in their ability to express themselves freely, probably since they felt their narrative to be accepted by the message. Accordingly, Interviewee No. 17 stated that she “*is not sure that the term ‘beasts’ is exactly something I would define, but sometimes maybe it’s even good to think that way to protect yourself*”. This change, albeit slight, forms the basis for the continued process of attitude and behavior change in the future.

#### 3.2.3. Third Segment

The majority of the *Initial Responses* after watching this segment, which conveyed the message “there is an alternative”, while briefly describing the history of the conflict between France and Algeria and noting that it was resolved peacefully, were characterized by positive emotions. Some participants even displayed a smile that they could not always explain, indicating the positive feelings the video evoked in them. Accordingly, 16 participants described experiencing positive emotions during this segment, particularly *hope* for future change in the context of the Israeli–Palestinian conflict and the possibility of resolving it peacefully. An analysis using AntConc revealed that the word “hope” was mentioned 29 times throughout all the interviews in the third segment. Interestingly, some participants expressed hope without explicitly using the word. For example, Interviewee No. 24 stated, “*There is a more ethical and moral option here that you hadn’t considered*”.

Even among those who were initially skeptical about peacemaking, there was an emotional shift in their response to the third segment. For instance, Interviewee No. 2, who expressed anger after watching the first segment, asserting that “there is no way out... and nothing will change”, described a sense of normalization after watching the third one. This led him to soften the anger, and he noted that the video instilled in him

*[…] a kind of hope, maybe each side can try to examine itself, do some self-reflection, think about how we can bridge the gap, maybe not in everything, but at least try to reach some common ground in some areas, and not see each other in such an extreme light as in the beginning.*

Additionally, other participants described feelings of joy and optimism, though at times these feelings were cautious and ambivalent, partly because they argued that the two conflicts could not be compared. For example, Interviewee No. 7 stated, “*I’m happy to hear it for them, but in our case, if you apply it to us, I’m not sure if it’s possible, but it’s encouraging, and I hope, maybe it will happen here too*”. Accordingly, an analysis using AntConc revealed that the word “joy” was mentioned by six different participants.

When examining *Openness or Closure* to new information, it seems that most participants were open to receiving the new information. Even those who rejected the message of this segment, as well as those who found it inspiring and agreed with it, were intrigued by the new information. They wanted to learn more and understand the causes of the conflict between France and Algeria and how it was resolved. This openness was accompanied by *cognitive ambivalence* regarding the applicability of the conclusions to the Israeli–Palestinian conflict. For example, Interviewee No. 18 stated:

*I agree that there is an alternative, but I think the comparison between this war and another war is a bit strange [...] because France had France, and it was perceived as a foreign entity that came to take over Algeria, while in Israel, it’s more of a war. I wouldn’t say an existential war, but it’s closer to home than what happened there.*

Overall, there is a clear relation between the type of emotions expressed by the participants and their *Acceptance or Rejection* of the message. The majority of the participants, 15 in total, described a positive emotional response to this message and accepted it to some extent. Their increased sense of openness and flexibility, reflected by their heightened cognitive ambivalence, also found among participants who watched IPM videos in Study 1, further supports previous research on the effects elicited by hope (e.g., [60],). Conversely, most of the participants who expressed a negative emotional response after watching this segment rejected the message and the justifications presented in the video for a peaceful resolution, arguing that there is no political solution to the Israeli–Palestinian conflict:

*Now, when I see it as a whole, it’s like the hypocrisy, the emotion with which it’s painted and fawning, overshadows all the other feelings I had before because it really leads you to this place of ‘look at these terrible things that were said, look at these terrible things that were done, see, it doesn’t have to be like this, and conflicts can be resolved differently’ and I’m like: ‘enough already’. (Interviewee No. 23)*

Viewing the third segment led to a *Change* in many participants, although its nature was varied. For some, the change was clearly reflected in a shift from the *precontemplation* stage to the *contemplation* stage. For example, Interviewee No. 5, who identified with the basic need to use delegitimization and argued that “*if someone comes to kill you, you should kill them first*”, described how watching the video caused her to somewhat question her existing stance and feel a desire to delve deeper and investigate to better understand the issue: “*[The video] made me wonder if maybe there really is another way, maybe things happened there that their handling can teach us how to act differently, how to think differently, optimism, it was like an optimistic ending*”. For others, *cognitive* and *emotional ambivalence* was evident, but it did not yet lead them to change their attitude about the Israeli–Palestinian conflict. For example, Interviewee No. 11 acknowledged the message as meaningful but found it difficult to apply the information to the Israeli–Palestinian conflict, noting its relevance to other conflicts in which Israel is involved:

*Yes, you can see optimism that conflict resolution can indeed happen, for example, at least in Israel, with countries that we don’t share a border with [...] [in the Israeli–Palestinian conflict] there is no optimism, there is pessimism and the thought that I won’t live to see it.*

For some participants, the change was reflected in a desire to deepen their knowledge and read more about the France–Algeria conflict to learn from it and develop the ability to draw conclusions and solutions in the context of the Israeli–Palestinian conflict. In other words, watching the video did not lead to an immediate change in their attitudes, but led them to contemplation, sparking their curiosity to learn more about the topic and broaden their range of arguments, similar to the reactions we found in Study 1. As Interviewee No. 2 noted:

*The third video, the short one, it presents a way of thinking, maybe trying to think differently, maybe trying to see how we can minimize this thinking, these words, these harsh things, look, it happened there, maybe it can happen in other places, maybe it’s a case study that can also help us, apply it to us.*

Thus, it seems that the *unfreezing* process has begun, and there is a heightened understanding that a shift in thinking is needed to consider alternative ways to resolve the conflict.

Conversely, there were participants for whom watching the third segment reinforced their existing attitude while expanding the arguments in its favor. For example, Interviewee No. 17, who supports resolving the Israeli–Palestinian conflict peacefully and felt uncomfortable during the previous segments, now sharpened her stance on these statements, noting that “*I wouldn’t want people to think extreme things about the Palestinian people or something like that, I also sometimes think extreme things, but in the end, I would be happy if it could be different, on both sides*”. Interviewee No. 4, who before watching did not believe that the Israeli–Palestinian conflict could be resolved peacefully, used the justification for the use of delegitimization to strengthen his arguments, stating, “*I don’t see any horizon [...] the demonization of the other side is necessary to maintain the situation or to survive*”.

#### 3.2.4. The Complete Video

The *Initial Response* among many participants after watching the entire video was to summarize their experience with optimism, describing the hope that we might live in peace side by side, similar to other countries that were once in conflict. Additionally, many participants mentioned that the video sparked considerable curiosity and a desire to explore the information presented to them. Others provided detailed descriptions of the emotional and cognitive transformation they underwent throughout the video, from sadness, anger, and even reminders of daily fears associated with the conflict, to relief, hope, and a new pathway of thought. Interviewee No. 15 described how watching the entire video made her reflect:

*It made me think that the structure of the video, the way it starts with emotions like anger and strong, harsh words, but in the end, it kind of reaches a conclusion of acceptance, of the idea that things can be different, and that it is possible, and not something that hasn’t happened before.*

On the other hand, a few participants described how watching the full video made them angry, particularly at what they perceived as an attempt to manipulate them, leading to resistance to change: “*From beginning to end, it felt very provocative, like it was trying to apply some sort of psychological manipulation that isn’t right for us, specifically regarding our conflict*” (Interviewee No. 7).

Regarding *Openness or Closure*, no new information was presented to the participants at this stage. It was evident that those who were open to new information during the various segments maintained their stance while watching the entire video. Similarly, in terms of *Acceptance or Rejection* of the message, most participants remained consistent with their previous positions; those who accepted the message in earlier segments generally became more curious to explore the conflict between France and Algeria and its resolution, and vice versa.

At this stage, the information processing experienced by the participants became more clear and deeply apparent: the sense of normalization of the previous messages was reinforced, and awareness of the process they were undergoing increased, leading some to accept the overall message. Interviewee No. 24 clearly noted the acceptance–change dialectic message, highlighting her sense of being accepted and understood (see [28]):

*I feel like someone understands me and empathizes with me, and along with that, at the end, after the message of ‘we understand your side’, there’s the idea that things can be different because it’s already happened somewhere else, and they’ve found a solution. It’s like connecting with the other person’s opinion and then asking them, ‘maybe you can ask yourself’—maybe something else is possible.*

As for *Change*, participants who had previously supported a peaceful resolution to the conflict did not move from their stance, with some even strengthening their position. However, it is noteworthy that some of them experienced a shift in their views regarding the necessity and use of the theme of delegitimization. After watching the video, they were better able to empathize with the message of the first and second segments, understanding that the use of this narrative is natural and normal. For example, Interviewee No. 14 said:

*When I watch it from beginning to end, certain things become clearer to me. For instance, at the beginning, when they said ‘we had to call them human beasts, we had to say they were murderers’, to maintain the narrative that the enemy is evil, suddenly it became very clear to me.*

Additionally, other participants who were convinced that peace was impossible before watching the video, remained steadfast in their position by the end. It appears that the brief video had no real impact on them, as their views were so firmly established that they remained *precontemplators*. For instance, Interviewee No. 21 stated that the video “*didn’t move me at all*” and that “*it feels more like they’re trying to disrupt my thinking and show me a different reality*”.

On the other hand, participants who were somewhat skeptical about the possibility of finding a solution to the Israeli–Palestinian conflict before watching the video reported going through a cognitive and emotional process after watching the entire video. This process led them to reconsider their positions and think that perhaps a solution is possible, accompanied by feelings of optimism (see [68]). For example, Interviewee No. 15 described the transition from the *precontemplation* stage to the *contemplation* stage:

*It raises questions for me, like how they did it, what their way of doing it was, if there was a third party involved […] So many questions about how it happens, because ultimately, that’s also my hope—that it will happen. I think the video really gave me some understanding that there might be certain ways they did it, so maybe it also applies to us, maybe not, but we should definitely know these methods and explore them a bit.*

Others described the ambivalent feelings and thoughts the video aroused in them, while expressing the unfreezing process they underwent regarding their perception of the Israeli–Palestinian conflict:

*Personally, it made me go through various emotions, from anger to firm agreement and amusement, and suddenly it made me change my thinking. I don’t know if ‘change’ is the right word, but it just made me look at it from a different angle... I think it made me think more that a solution might be possible. (Interviewee No. 17)*

### 3.3. Discussion

Study 2 aimed to explore the expressions of cognitive and emotional ambivalence following exposure to different segments of an IPM-based video, as well as assess their potential influence to facilitate contemplation. The findings of the study indicated that the *acceptance* parts of the video (first and second segments), introducing and normalizing the theme of delegitimization mostly created negative emotions of anger and fear, commonly found among members of societies involved in intractable conflicts (see [57]). However, alongside ambivalent feelings and discomfort, they created growing interest and gradually allowed many participants to openly identify with their message, as it probably created a sense that their narrative is being accepted.

The *change* part of the video, presenting the way the French–Algerian conflict ended peacefully, intensified the ambivalent feelings and thoughts about the comparison between the conflicts, generating much curiosity among the vast majority of the participants. It also predominantly created relief and elicited hope, thus leading those participants to accept the message that there is potential for peaceful resolution. Moreover, the ones who experienced at this stage positive emotions clearly demonstrated contemplation through expressing willingness to reconsider their perspective about the Israeli–Palestinian conflict, as well as to learn more about the topic and broaden their range of arguments. Conversely, fewer participants expressed negative feelings of anger at this stage, and even though some were curious and open about the new information provided, they rejected it and stayed in precontemplation.

The qualitative inquiry of participants’ reactions to the IPM-based intervention provided a unique opportunity for a systematic look at the transition from precontemplation to contemplation that it enables. Although not all participants changed their perspective about the possibility to peacefully resolve the Israeli–Palestinian conflict, a large number engaged in a process characterized by cognitive and emotional ambivalence. The IPM-based intervention created a sense of acceptance for participants, which allowed them to gradually engage with the newly provided information about possible change. The increased thoughtful and emotional engagement motivated many to reevaluate and reflect on their current perspective about the conflict.

## 4. General Discussion

The findings from both studies exemplify some of the IPM’s suggested advantages over other interventions. The IPM-based intervention did not provide only information that contradicted participants’ existing attitudes and beliefs, but rather provided information in a dialectic manner, presenting validation alongside challenges to conflict-supporting attitudes and beliefs. Accepting and challenging conflict-supporting beliefs, in tandem with the general context of intractable conflicts, may have afforded participants the opportunity to be more open to reconsidering their current perspective. The dialectic approach allowed them to reflect about reasons to keep or change their perspective in ways that most resonate with them, which is a key component for lasting change [35,89]. An additional benefit of the IPM’s dialectic method is that it facilitated participants’ increased understanding of the attitudes and beliefs of those on the other side of the political spectrum. This potential benefit of allowing greater understanding and decreasing polarization within one’s own group is of special importance during peace processes, when social polarization intensifies and threatens their viability and continuation [90].

Additionally, Study 1 found that participants in the IPM condition engaged in greater deliberation of the message contents compared to the control condition. Study 2 provided detailed knowledge about the cognitive and emotional aspects of this deliberation, thus reflecting our explanatory bi-directional approach to merging the two analyses [52]. The finding that IPM-based interventions successfully stimulate deeper thinking and reflection on the conveyed messages, particularly in the context of the Israeli–Palestinian conflict, is also consistent with previous studies [7,28]. Furthermore, beyond affirming the effects of IPM-based interventions, our research provides insights into the underlying process. Therefore, it seems that these interventions are effective through the creation of a heightened sense of ambivalence and a transition from the precontemplation to the contemplation stage. These findings highlight the importance of considering the change of attitudes and beliefs in intractable conflicts as a gradual process. That way, we can observe progress toward change before we see actual change, rather than potentially dismissing interventions as wholly ineffective.

We also found in both studies that some of the participants were not only showing that they were ready to reconsider their beliefs, but also expressed that they were looking for ways to make the change. Integrating the findings from both studies, the latter could be interpreted as a characteristic of preparation, which is marked by a desire for taking action, but not knowing quite how to initiate that change [41]. In the context of intractable conflict-related attitudes and beliefs, preparation could be described as the stage in which one acknowledges that their existing attitudes and beliefs are costly, and while they desire to adopt alternative attitudes and beliefs that are less costly, they are unsure of what those might be. Action could be described as the stage in which one is actively taking steps to modify or replace their previously held attitudes and beliefs. Implementing this next step by developing interventions to support transitions to more advanced stages of changing attitudes and beliefs in intractable conflicts can be explored further in future studies.

It is worth recalling that the two studies were carried out prior to the October 7th attack on Israel and the current Israel–Hamas war. As surveys preformed since the beginning of the war point to a sharp decline in support for negotiation and compromises among Jewish–Israelis [1,3], one can assume that fewer people are now in the preparation and contemplation stages and more people moved back to precontemplation regarding their conflict-related narrative and attitudes. Our findings emphasize the need to create ambivalence among precontemplators to begin the process of unfreezing conflict-supporting narrative. Based on our current and previous studies, carried out in the context of violent conflict, we suspect that wide-scale application of IPM-based interventions could spark ambivalence and initiate this gradual process. Testing this assumption requires further empirical work.

### Limitations and Future Directions

It is important to note that it is challenging to assess practical and long-term behavioral changes within the context of a brief questionnaire or interview. Therefore, we focused, in both studies, on the potential to influence changes in the participants’ attitudes rather than behavior.

Another limitation that comes with studying attitude and belief change as a gradual process is that the research itself requires a slow and gradual process. The TTM is a robust model that has been effectively adapted to many new contexts, including social psychology (e.g., [43,91,92]), organizational change (e.g., [44]), the criminal justice system [42], technology adoption [93], and financial behavior [94]. However, attempting such change in the context of intractable conflicts comes with its own challenges. For example, considering that conflict supporting attitudes and beliefs serve important psychological and social functions—including providing a sense of identity, security, certainty, social cohesion, and meaning [8]—it seems unrealistic and possibly even inappropriate to attempt to change those attitudes abruptly, or in a short period of time. Even if possible, it is not at all obvious that suddenly reversing an attitude or belief that provides a sense of identity, ontological security, and/or meaning, in the context of intractable conflicts, is more beneficial than harmful (see [95]). Therefore, this research incorporates the TTM as an established model that facilitates lasting change as a gradual process, recognizing the deeply rooted nature of these attitudes and beliefs while providing a framework for sustainable transformation over time. But doing so effectively and ethically requires the utmost sensitivity to those under the researchers’ gaze.

An additional limitation of this study is that it only involved Jewish Israelis. While one of our previous studies found initial support for the IPM principles among a representative sample of Palestinians in the Occupied Territories ([7], Study 1), it is important to examine IPM-based interventions among this group. The asymmetrical nature of the Israeli–Palestinian conflict, taking a much higher human toll from the low-power Palestinian group (see https://statistics.btselem.org/en/ (accessed on 1 October 2024)), creates different perspectives on its manifestations and required conditions for its resolution (e.g., [96,97]). Therefore, future studies should examine whether these differing statuses and perspectives create differences in the psychological mechanisms and outcomes of IPM-based interventions aimed at promoting peaceful attitudes.

Combining research from social and clinical psychology offered us a valuable framework and a more holistic perspective for understanding and facilitating attitude change, particularly in the challenging context of intractable conflicts. First, recognizing attitude change as a process, which is common in clinical psychology (e.g., [98]), rather than an immediate outcome was helpful, as conflict-related attitudes are often deeply entrenched and resistant to change [8,39]. This theoretical integration allowed us to make the important focal shift from results to processes. Hence, the TTM is integral to deepening our understanding of changing attitudes, and acts as a stepping stone for similar endeavors in the future. This research also allowed us to leverage existing therapeutic techniques that address deeply ingrained beliefs and promote gradual shifts in perspective, such as the acceptance–change dialectic, which contributed to developing the IPM.

In conclusion, conflict-supporting narratives contribute to the continuation of violent conflicts, which exact great costs from the involved societies in terms of human lives, well-being, and property. Due to the deep entrenchment of such narratives, it is of crucial importance not only to develop interventions to unfreeze them, but to understand *how* such interventions exert their effects. The present research takes us a step forward in understanding the processes underlying the effects of IPM-based interventions. Although we focused on the Jewish–Israeli side within the context of the Israeli–Palestinian conflict, the IPM-based intervention is not conflict-specific in its content. Furthermore, the cognitive and emotional processes found in the current research facilitate contemplation, which is a human and universal change process. Future work might utilize this comprehensive understanding of the IPM in order to refine it and adapt it to other contexts. This could potentially contribute to advancing peaceful conflict resolution and reducing human suffering not only among Israelis and Palestinians, but elsewhere as well.

## Data Availability

The data presented in Study 1 are openly available in OSF at https://osf.io/a32kj/?view_only=00fd3eb506a342cd8a3efa5982391b70 ( accessed on 1 October 2024). The data presented in Study 2 can be obtained from the authors on request.

## Supplementary Materials

The following supporting information can be downloaded at: https://www.mdpi.com/article/10.3390/bs14121152/s1, Table S1: Open-ended Attitudinal Balance in Conflict, Study 1; Table S2: Kappa Scores of Interrater Reliability for Each Theme from Study 1; Table S3: Descriptive Statistics and Analysis of Thematic Responses, Study 1; Table S4: Descriptive Statistics and T-tests of the IPM-based Intervention on Thematic Codes, Study 1; Table S5: Examples of Thematic Responses by Reasons to Change and Maintain Stances, Study 1; Table S6: Interview Protocol, Study 2.
