# Supplementary material for: Understanding Reactions to Informative Process Model Interventions: Ambivalence as a Mechanism of Change
Source: Behav Sci (Basel). 2024 Dec 2;14(12):1152. doi: 10.3390/bs14121152 (PMC11672873; doi:10.3390/bs14121152)
Supplement: Supplementary file 1 [file behavsci-14-01152-s001.zip › behavsci-3264746-supplementary.pdf]

Supplementary Materials

Table S1: Open-ended Attitudinal Balance in Conflict, Study 1

Think of as many reasons for each decision as you can. Then rate the arguments for each decision from what you find most compelling to least compelling.

Decision	For	Against
To Reassess my political opinions about the Israeli-Palestinian conflict	Reason 1<br>Reason 2<br>Reason 3<br>Reason 4<br>Reason 5<br>Reason 6	Reason 1<br>Reason 2<br>Reason 3<br>Reason 4<br>Reason 5<br>Reason 6
To maintain my current political beliefs and understandings about the Israeli-Palestinian conflict.	Reason 1<br>Reason 2<br>Reason 3<br>Reason 4<br>Reason 5<br>Reason 6	Reason 1<br>Reason 2<br>Reason 3<br>Reason 4<br>Reason 5<br>Reason 6

Note: Participants were required to write at least one reason for each of the 4 prompts. After which, an additional space appeared for them to add additional reasons if they had them. Each of the 4 prompts allowed up to 6 reasons to be entered.

Table S2: Kappa Scores of Interrater Reliability for Each Theme from Study 1

**Dependent Measure**	***Kappa***
Personal Serenity	.668
Ingroup Focused Reasoning	.767
Outgroup Focused Reasoning	.940
Collective Focused Reasoning	.603
Seeking to Influence Others	.760
Peer Pressure	.923
**No Response**	**.921**
Peace	.928

Unity	.650
Positive Emotions	.842
Negative Emotions	.746
Positive Personal Experience	.835

Note: The themes and scores that are highlighted in bold are the ones that were statistically significant between conditions.

Table S3: Descriptive Statistics and Analysis of Thematic Responses, Study 1

Response Themes	Condition	Percent of Participants	df	χ^2	p
Having or Wanting Personal Serenity	IPM	12.9%	1	0.23	.635
	Control	10.9%			
Considerations Centered Around Participant's Ingroup	IPM	40.5%	1	1.88	.109
	Control	31.9%			
Considerations Centered Around Participant's Outgroup	IPM	51.7%	1	2.22	.136
	Control	42.0%			
Considerations Centered Around a Collective Focus (Ingroup & Outgroup Together)	IPM	34.5%	1	0.28	.595
	Control	37.8%			
Participant's Ability or Desire to Influence Others' Perspectives	IPM	15.5%	1	1.19	.276
	Control	21.0%			
Participant's Desire to be Accepted by Others as an Influencing Factor	IPM	10.3%	1	.081	.776
	Control	9.2%			

**Participants Not Responding, Because They Cannot or Will Not Think of Considerations**	**IPM**	**31.0%**	1	**4.02**	**.045**
	**Control**	**43.7%**			
Seeking or Wanting Peace	IPM	31.0%	1	0.00	.992
	Control	31.1%			
Seeking or Wanting Unity	IPM	8.6%	1	0.35	.552
	Control	10.9%			
Expressing Positive Emotions Relating to their Considerations	IPM	27.6%	1	1.78	.182
	Control	20.2%			
**Expressing Negative Emotions Relating to their Considerations**	**IPM**	**19.8%**	**1**	**5.32**	**.021**
	**Control**	**9.2%**			
Seeking, or Having Positive Personal Experiences	IPM	18.1%	1	0.32	.575
	Control	21.0%			
Expecting, or Having Negative Personal Experiences	IPM	17.2%	1	0.07	.793
	Control	16.0%			
Seeking, or Having Positive Interpersonal Experiences	IPM	34.5%	1	0.28	.595
	Control	37.8%			
Expecting, or Having Negative Interpersonal Experiences	IPM	37.1%	1	2.80	.094
	Control	26.9%			
**Government and Statehood**	**IPM**	**41.4%**	1	**7.12**	**.005**
	**Control**	**24.4%**			
Thinking that the Conflict May Not, or Will Not, Change	IPM	13.8%	1	1.20	.274
	Control	9.2%			

**Jewish Identity as a Factor**	**IPM**	**19.8%**	**1**	**6.35**	**.012**
	**Control**	**8.4%**			
Responses with Qualifiers	IPM	37.9%	1	0.81	.369
	Control	43.7%			
**Aggression and Violence**	**IPM**	**32.8%**	**1**	**4.79**	**.029**
	**Control**	**20.2%**			
Safety and Security	IPM	26.7%	1	2.28	.131
	Control	18.5%			
Impacts on Economy	IPM	18.1%	1	3.13	.077
	Control	10.1%			
Land and Territory	IPM	22.4%	1	0.04	.839
	Control	23.5%			

Note: The themes and scores that are highlighted in bold are the ones that were statistically significant between conditions.

Table S4: Descriptive Statistics and T-tests of the IPM-based Intervention on Thematic Codes, Study 1

Measure	IPM (N=116)		Control (N=119)						95% CI	
	M	SD	M	SD	DF	t	p	Cohen's d	Lower Bound	Upper Bound
Having or Wanting Personal Serenity	0.03	0.12	0.02	0.07	233	0.67	0.51	0.09	-0.02	0.03
Considerations Centered Around	0.08	0.12	0.08	0.15	233	-0.11	0.91	-0.01	-0.04	0.03

Participant's Ingroup										
Considerations Centered Around Outgroup	.015	0.19	0.12	0.17	233	1.28	0.20	0.17	-0.02	0.08
Considerations Around Collective Groups	0.09	0.17	0.09	0.15	233	0.29	0.77	0.04	-0.03	0.05
Participant's Ability or Desire to Influence Others' Perspectives	0.04	0.11	0.06	0.13	233	-1.45	0.15	-0.19	-0.05	0.01
Participant's Influenced by Desire to be Accepted by Others	0.02	0.06	0.02	0.08	233	-0.49	0.62	-0.06	-0.02	0.01
Participants Cannot or Will Not Think of Considerations	0.11	0.21	0.19	0.28	233	-2.49	0.01	-0.32	-0.15	-0.02
Seeking or Wanting Peace	0.08	0.16	0.07	0.13	233	0.50	0.62	0.06	-0.03	0.05
Seeking or Wanting Unity	0.01	0.05	0.02	0.06	233	-0.61	0.55	-0.08	-0.02	0.01
Expressing Positive Emotions Relating to their Considerations	0.06	0.13	0.05	0.10	233	0.89	0.38	0.12	-0.02	0.04
Expressing Negative Emotions Relating to their Considerations	0.04	0.09	0.01	0.05	233	2.81	0.01	0.37	0.01	0.05
Seeking, or Having Positive	0.03	0.09	0.04	0.09	233	-0.55	0.59	-0.07	-0.03	0.02

Personal Experiences										
Expecting, or Having Negative Personal Experiences	0.03	0.07	0.03	0.08	233	-0.27	0.79	-0.03	-0.02	0.02
Seeking, or Having Positive Interpersonal Experiences	0.07	0.11	0.09	0.14	233	-1.57	0.12	-0.20	-0.06	0.01
Expecting, or Having Negative Interpersonal Experiences	0.07	0.12	0.06	0.11	233	1.27	0.21	0.16	-0.01	0.05
Government and Statehood	0.11	0.18	0.07	0.15	233	2.17	0.031	0.28	0.00	0.09
Thinking that the Conflict May Not, or Will Not, Change	0.03	0.07	0.02	0.08	233	0.35	0.724	0.05	-0.02	0.02
Jewish Identity as a Factor	0.04	0.11	0.03	0.19	233	0.57	0.5697	0.07	-0.03	0.05
Responses with Qualifiers	0.11	0.19	0.16	0.25	233	-1.71	0.09	-0.22	-0.11	0.01
Aggression and Violence	0.07	0.13	0.04	0.09	233	2.19	0.03	0.29	0.00	0.06
Safety and Security	0.05	0.10	0.05	0.11	233	0.27	0.79	0.04	-0.02	0.03
Impacts on Economy	0.04	0.10	0.02	0.07	233	1.70	0.09	0.22	0.00	0.04
Land and Territory	0.05	0.11	0.06	0.14	233	-0.54	0.59	-0.07	-0.04	0.02

Table S5: Examples of Thematic Responses by Reasons to Change and Maintain Stances, Study 1

Theme	Reason	Example Consideration Implications
**Government/Statehood**	Change	"Less government funds will be wasted"<br>"We will have a more diverse state"<br>"The global attitude towards Israel will change for the better"
	Maintain	"To preserve political stability"<br>"Supporting the state of Israel"<br>"The state of Israel will remain ours"
**Aggression/ Violence**	Change	"Maybe fewer people will be killed, maybe not"<br>"Less rigid policies may lead to more terrorist attacks"<br>"It would be possible to live quietly in our country without terrorist attacks"
	Maintain	"To reduce the amount of terrorist attacks"<br>"To maintain the ability to denounce violence from any side of any kind"<br>"Avoiding the death of soldiers and civilians from both sides"
**Negative Emotions**	Change	"Unnecessary hatred"<br>"To live with less anger and resentment towards the other side"<br>"If we continue to see the enemy as a friend we will experience an unpleasant surprise when they rise up against us more organized"
	Maintain	"Not to live in fear"<br>"Hatred will increase"<br>"Maybe I can influence others to value of peace over hatred"

**Jewish Identity**	Change	"A rift between the Jewish people"<br>"Ensuring the preservation of the Jewish character of the State of Israel"<br>"Confidence in the integrity of the Jewish people"
	Maintain	"We may lose our right as Jews to settle in the Jewish state"<br>"Loyalty to Jewish identity and historical territorial rights"<br>"That I may preserve my Jewish identity"
**Nothing**	Change	"I don't think I might lose if my positions remain the same"<br>"I do not know…"<br>"Not too much…"
	Maintain	"It's hard for me to answer that"<br>"Nothing"<br>"It doesn't matter what my positions are"
**Influencing Others**	Change	"Changing my, and others' thoughts we'll get to know the other sides"<br>"The change to change someone else's opinions"<br>"I would make it harder for people to stay in their bubble"
	Maintain	"I may have a social impact"<br>"I would lose my voice at the ballot box"<br>"If I change, there will be a greater rift in society"
**Ingroup Focus**	Change	"I may lose people who disagree with me"<br>"I would get a stronger bond with my father"<br>"I would argue less with friends"

	Maintain	"If my opinions do not change, I will continue to visit my family"<br>"My mother will remain satisfied"<br>"My group affiliation"
**Outgroup Focus**	Change	"Find a compromise that will also fit their side"<br>"Live with less anger and resentment towards the other side"<br>"The Arab public will gain more support"
	Maintain	"The territories that the Palestinians want will remain in our hands"<br>"Knowing a culture different from mine"<br>"Good relations with the Arabs"
**Safety/ Security**	Change	"Fewer soldiers and a smaller army"<br>"A sense of security"<br>"Less investment in security and more investment in other areas"
	Maintain	"Unrest, political instability and insecurity"<br>"Better control over the borders and crossings"<br>"Deterrent Power"
**Land**	Change	"An incessant and inconclusive struggle for control of Israel"<br>"Territories that belong to the Land of Israel"<br>"Safeguarding Israeli territories"
	Maintain	"If my opinions do not change, I will help settle the country"<br>"We will gain a connection to our heritage and our right to the land"<br>"Annexation of territories and reduction of borders"

Table S6: Interview Protocol, Study 2

Stage 1: Introduction	The participant is reminded that the interview is being recorded and that they have given their consent, as indicated on the form signed prior to the interview. To create a relaxed atmosphere, the interviewer briefly introduces themselves and asks the participant to briefly talk about themselves. The following questions, among others, are asked for this purpose:<br><br>1. How old are you?<br>2. Where in the country are you from?<br>3. Where do you study, and for which degree?<br>4. Where did you serve in the military?<br>5. Tell me briefly about your family.<br>6. How would you define your political engagement? Who influenced your views?<br><br>Following this, the participant is provided with a brief explanation of the research process without revealing any critical information: "I will now show you a short video, after which we will have a discussion. Please watch the video carefully. If there are any issues with the connection, or for any reason you cannot view the video, please let me know." After addressing any questions, the interviewer shares their screen and plays, in stages, different segments of the video.
Stage 2: Post-Viewing Discussion	After viewing each segment, the interviewer ensures that the participant watched the video without interruptions and then asks the relevant questions according to the segment.<br><br>**Questions for Segment 1:** A. Please describe how you feel when you hear these statements. B. Please specify what thoughts arose in your mind when you heard these statements.<br><br>**Questions for Segment 2:** A. Please describe how you feel when you hear these statements. B. Please specify what thoughts arose in your mind when you heard these statements. C. Initially, what conflict did you think the video was addressing? How do you feel now after the initial viewing and the reveal of the character in the image?

	**Questions for Segment 3:** A. Please describe how you feel when you hear these statements. B. Please specify what thoughts arose in your mind when you heard these statements.<br><br>**Questions after viewing the entire video:** A. What did you think of the video? B. Was the video surprising in any way? If yes, how so? C. Did the video provide you with any new information that you were not previously aware of? If yes, what was it? D. Did the video lead you to understand things you had not realized before? If so, what, for example? E. Did you find any connection or similarity between the video and other events you have experienced in your life? If so, in which segment? F. What emotions and thoughts did the video evoke in you? Which part of the video triggered those feelings? G. Did the video impact your stance, even slightly, on the Israeli-Palestinian conflict, particularly regarding the possibility of finding a resolution? H. After watching the video, did any new ideas for peacefully resolving the Israeli-Palestinian conflict arise in your mind? If yes, what ideas emerged—these could be original and creative ideas that had not been considered before.
Stage 3: Conclusion	The participant is thanked for their participation and significant contribution to the research.
